# Effects of Quality Control Targets (SpO2≠100%, PaCO2/<40 mmHg, Pmean/>10 cmH2O) on Outcomes in Patients in the ICU

**DOI:** 10.3389/fmed.2020.00111

**Published:** 2020-04-15

**Authors:** Pan Pan, Longxiang Su, Qing Zhang, Yun Long, Xiaoting Wang, Dawei Liu

**Affiliations:** ^1^Department of Critical Care Medicine, Peking Union Medical College Hospital, Peking Union Medical College & Chinese Academy of Medical Sciences, Beijing, China; ^2^Center of Respiratory and Critical Care Medicine, Chinese PLA General Hospital, Beijing, China

**Keywords:** quality control targets, hyperoxia, lung and circulation protective ventilation, outcome, critically ill patients

## Abstract

**Objectives:** A series of quality control (QC) targets (SpO2≠100%, PaCO2≮40 mmHg, Pmean≯10 cmH2O) was put forward and widely used in a single intensive care unit (ICU) setting. The aim of this study was to assess whether these QC targets could improve the outcomes of critically ill patients.

**Methods:** The real-time clinical data of patients undergoing mechanical ventilation at ICU admission between May 2013 and May 2017 in the Department of Critical Care Medicine of Peking Union Medical College Hospital were collected and analyzed.

**Results:** A total of 7,670 patients [mean age, 58 years; 3,943 (51.5%) male] were divided into the before QC (*n* = 3,936) and after QC (*n* = 3,734) groups. QC targets (SpO2, PaCO2, and Pmean) and respiratory parameters (FiO2%, PaO2, PEEP, tidal volume, and respiratory rate) within 72 h of ICU admission, primary outcomes (ICU mortality, 28-, 60-, and 90-day mortality) and secondary outcomes (discharge against medical advice, ICU admission days, mechanical ventilation times, and central venous pressure) were measured and compared between the before and after QC groups. The 72 h average of the Pmean, FiO2%, PaO2, and VT were significantly lower and PaCO2 was higher in the after QC than in the before QC group (*P* < 0.05). A lower 90-day mortality rate, less discharge against medical advice, fewer ICU admission days, and reduced mechanical ventilation times were found in the after QC group compared with the before QC group (*P* < 0.05). Interestingly, CVP was significantly lower in the after QC group than in the before QC group (*P* < 0.05).

**Conclusions:** The QC targets (SpO2≠100%, PaCO2≮40 mmHg, Pmean≯10 cmH2O) contributed to avoiding high oxygen level hazards, protecting against lung injury, and improving circulatory function, which resulted in a better prognosis of critically ill patients.

## Introduction

With the development of intensive care, clinicians are increasingly aware of the important role of some therapeutic concepts in critically ill patients, such as the assessment and implementation of fluid responsiveness, the use of lung protective ventilation, and the control and prevention of catheter-related infection. In daily clinical work, clinicians use these concepts to treat critically ill patients and achieve satisfactory results, accompanied by a decreased mortality rate. Therefore, how these proven clinical practices can be used to control certain indicators to achieve the purpose of treatment drew our attention. Intensive care involves many measurements and monitoring indicators; however, treatment measures are different, and due consideration has to be given to ways in which to improve the quality of intensive care.

Breathing and circulation are the primary problems that need to be addressed in critically ill patients. Most critically ill patients need to undergo invasive mechanical ventilation while in the intensive care unit (ICU). However, invasive mechanical ventilation is different from physiological respiration. Improper use of mechanical ventilation may result in lung injury, resulting in increased treatment failure and mortality (1). Oxygenation, maintaining ventilation, and achieving ideal pulmonary gas exchange are particularly important. Practice has proven that high oxygen levels are harmful (2, 3). The need for a small tidal volume in lung protective ventilation has been recognized (4, 5). The impact of mechanical ventilation on the circulation has increasingly become a point of concern and a research hot spot (6–8). In our daily work, three parameters are used to control the effects of mechanical ventilation on breathing and circulation in critically ill patients: SpO2, PaCO2, and Pmean. SpO2 is used to control the concentration of oxygen inhaled. PaCO2 and Pmean are used to control the lung injury caused by mechanical ventilation. Furthermore, Pmean also plays a role in the impact of ventilation on circulatory functioning (9, 10). These three clinical targets are used to achieve the goal of treatment and reduce mortality. This study investigated the use of these three quality control (QC) targets before and after targets were implemented and showed that respiratory and circulatory QC targets affected the prognosis of critically ill patients.

## Methods

### Patient Sample

We performed a retrospective study among all patients who were admitted to the Department of Critical Care Medicine, Peking Union Medical College Hospital from May 2013 to May 2017. This study included all patients with tracheal intubation and mechanical ventilation upon admission to the ICU. Patients without invasive mechanical ventilation were excluded. The Institutional Research and Ethics Committee of the Peking Union Medical College Hospital approved this study for human subjects.

### QC Targets and Directly Relevant QC Parameters

**Figure 1:**
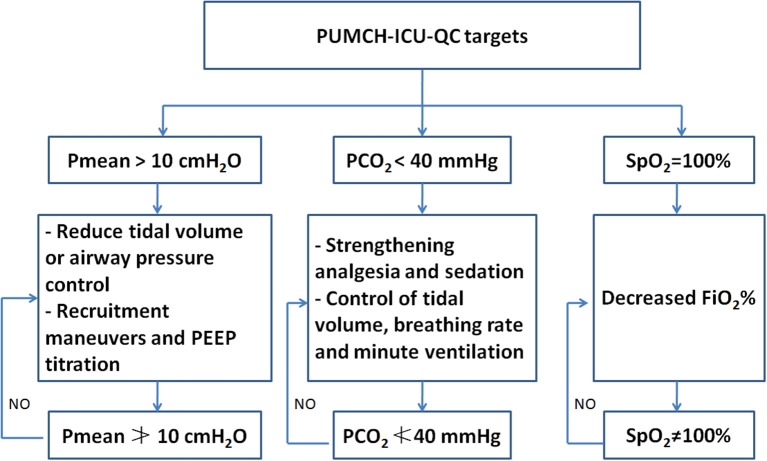
The brief description of the strategy and protocol used to adjust SpO2, PaCO2, and Pmean. PUMCH, Peking Union Medical College Hospital; ICU, intensive care unit; QC, quality control.

Three parameters (SpO2, PaCO2, and Pmean) were employed and used as quality targets during treatment after July 2015 based on our clinical experience. The target values of SpO2, PaCO2, and Pmean were set to SpO2≠100%, PaCO2≮40 mmHg, and Pmean≯10 cmH2O. The directly relevant QC parameters were defined as the parameters that contribute to achieving those targets, such as FiO2, PaO2, PEEP, VT, and RR, and were set based on the lung-protective ventilation strategy (11). The strategy and protocol used to adjust these three targets are described in Figure 1. We used these three targets after July 2015. Therefore, all the patients admitted to the ICU after July 2015 were included in the after QC group (July 2015–May 2017). Correspondingly, the patients admitted between May 2013 and June 2015 were included in the before QC group.

### Mechanical Ventilation Mode and Pmean Measurement

Lung protective strategies for mechanical ventilation were used with all of the patients who were admitted to the ICU (11). When the patients were under adequate sedation and analgesia but lacked spontaneous breathing, volume-controlled or pressure-controlled ventilation was used. Once the patient had spontaneous breathing, controlled ventilation was immediately converted to pressure support ventilation. Pmean is the average airway pressure over several breathing cycles, which is equal to the area under the pressure–time curve divided by the breathing cycle. Pmean not only represents the alveolar oxygenation state but also reflects the hemodynamic state. In the controlled ventilation mode, the following formula can be used to approximate the Pmean of each breath:

$$\begin{array}{l} \text{Pmean}=\text{k}*\left(\text{Ppeak}-\text{PEEP}\right)*\text{Ti}/\text{Ttotal}+\text{PEEP} \end{array}$$

*K* is the coefficient (pressure-controlled ventilation is 1, volume-controlled ventilation is 0.5), Ti is the inspiratory time, and Ttotal is the breathing cycle.

Pmean is measured automatically by the mechanical ventilator.

### Data Collection

All of the clinical data came from the Peking Union Medical College Hospital Intensive Care Medical Information System (PICMIS), which records real-time monitoring data and treatment information from the bedside each hour. The first 72 h of clinical data from mechanical ventilation patients were recorded. The average, maximum, and minimum values were calculated in the database and exported into an Excel sheet. Some patients died or were transferred out of ICU within 72 h and others stayed in the ICU for more than 72 h. We used the available data for all the patients within the first 72 h.

### Statistical Analysis

Descriptive analysis was performed. All data are expressed as the means ± SD and were analyzed using *t*-tests. The baseline and outcome classification variables were compared with χ^2^ tests. All comparisons were two-tailed, and a *p* < 0.05 was required to exclude the null hypothesis. Statistical analyses were performed using the SPSS 18.0 software package (SPSS, Chicago, IL).

## Results

### Patients

During this study period (May 2013–May 2017), our ICU accepted 10,666 patients. A total of 2,996 patients without mechanical ventilation were excluded. The remaining 7,670 patients were divided into the before QC (QCB) and after QC (QCA) groups. Table 1 shows the characteristics of the patients at study inclusion. The age, sex, APACHE II score, and SOFA score of the patients before and after QC were not significantly different. Our ICU patients were mainly surgical patients.

**Table 1:** Characteristics of the patients at study inclusion divided into the QCB and QCA groups.

**Characteristics**	**Before Quality Control (QCB group)**	**After Quality Control (QCA group)**	***P*-value**
	***N* = 3,936**	***N* = 3,734**	
Age (years)	61 (47–72)	61 (48–72)	0.465
Sex (male/female)			0.234
Male (*n*, %)	2052 (52.1)	1896 (50.8)	
Female (*n*, %)	1884 (47.9)	1838 (49.2)	
APACHEII score	15 (10–21)	14 (11–19)	0.153
SOFA score	8 (5–11)	7 (5–10)	0.144
Type of ICU admission			0.064
Medical (*n*, %)	254 (6.5)	332 (8.9)	
Surgical (*n*, %)	3682 (93.5)	3402 (91.1)	
Compliance rate of quality control targets (*n*, %)	2654 (67.4)	3218 (78.8)	<0.001

*Quantitative data are expressed as the mean ± SD or median interquartile (25–75)%. Qualitative data are expressed as n (%)*.

### Targets Before and After QC and the Relevant Parameters

Table 2 shows the changes in SpO2, PaCO2, and Pmean before and after QC. The average and maximum SPO2 values were higher in the QCB group than in the QCA group. The average PCO2 of the QCA group was higher than that of the QCB group. The average and maximum Pmean values were lower in the QCA group than in the QCB group. Table 3 shows the relevant parameters of the changed QC targets. It shows that average, maximum, and minimum FiO2 values; PaO2 values; and the average, maximum, and minimum VT values were significantly lower after QC. Although the maximum and minimum PEEP values and the minimum RR differed before and after QC, the actual clinical significance may not be significant.

**Table 2:** QC targets before and after quality control.

**Characteristics**	**Before Quality Control (QCB group)**	**After Quality Control (QCA group)**	***P*-value**
	***N* = 3,936**	***N* = 3,734**	
SPO2 avg (%)	98.6 ± 3.1	98.3 ± 3.0	<0.001
PaCO2 avg (mmHg)	38.8 ± 5.3	39.5 ± 4.6	<0.001
Pmean avg (cmH2O)	8.8 ± 2.1	8.7 ± 1.7	0.009
SPO2 max (%)	99.9 ± 0.5	99.9 ± 0.7	0.011
PaCO2 max (mmHg)	44.4 ± 9.2	44.6 ± 8.2	0.182
Pmean max (cmH2O)	11.8 ± 4.9	11.4 ± 4.0	0.034
SPO2 min (%)	90.0 ± 18.4	90.9 ± 15.0	0.019
PaCO2 min (mmHg)	33.4 ± 5.7	34.0 ± 5.7	<0.001
Pmean min (cmH2O)	5.8 ± 2.9	6.2 ± 2.6	<0.001

*Quantitative data are expressed as the mean ± SD*.

**Table 3:** Relevant parameters before and after quality control.

**Characteristics**	**Before Quality Control (QCB group)**	**After Quality Control (QCA group)**	***P* value**
	***N* = 3,936**	***N* = 3,734**	
FiO2 avg (%)	39.9 ± 8.5	32.1 ± 9.1	<0.001
PaO2 avg (mmHg)	139.1 ± 36.8	112.3 ± 30.0	<0.001
PEEP avg (cmH2O)	5.3 ± 1.5	5.4 ± 1.3	0.304
VT avg (ml)	337.2 ± 149.6	321.9 ± 140.4	<0.001
RR avg (bpm)	17.1 ± 2.6	17.1 ± 2.5	0.448
FiO2 max (%)	52.7 ± 30.6	47.6 ± 34.8	<0.001
PaO2 max (mmHg)	193.6 ± 98.4	154.7 ± 66.1	<0.001
PEEP max (cmH2O)	6.3 ± 3.4	6.1 ± 3.3	0.006
VT max (ml)	395.3 ± 133.4	382.7 ± 136.9	<0.001
RR max (bpm)	27.3 ± 7.9	27.3 ± 8.1	0.868
FiO2 min (%)	35.4 ± 10.0	26.9 ± 8.8	<0.001
PaO2 min (mmHg)	101.2 ± 44.9	85.3 ± 32.3	<0.001
PEEP min (cmH2O)	4.5 ± 1.3	4.7 ± 1.2	<0.001
VT min (ml)	297.1 ± 180.8	280.0 ± 173.2	<0.001
RR min (bpm)	9.7 ± 4.2	10.0 ± 4.2	0.003

*Quantitative data are expressed as the mean ± SD*.

### Outcome Data

The outcome data are shown in Table 4. Although ICU mortality, 28-day mortality, and 60-day mortality were not significantly changed, discharge against medical services and 90-day mortality was substantially lower after QC. The number of ICU admission days was shortened, and the duration of mechanical ventilation was lower after QC. Interestingly, the average, maximum, and minimum CVP values were all significantly lower after QC than before QC.

**Table 4:** Outcomes before and after quality control.

**Characteristics**	**Before Quality Control (QCB group)**	**After Quality Control (QCA group)**	**Absolute Risk Reduction (95% CI)**	***P*-value**
	***N* = 3,936**	***N* = 3,734**		
ICU mortality (*n*, %)	74 (1.9)	73 (2.0)	−0.001 (−0.007–0.005)	0.811
Discharge against medical advice (*n*, %)	153 (3.9)	88 (2.4)	0.015 (0.007–0.023)	<0.001
28-day mortality (*n*, %)	138 (3.5)	122 (3.3)	0.002 (−0.006–0.010)	0.563
60-day mortality (*n*, %)	167 (4.2)	140 (3.7)	0.005 (−0.004–0.014)	0.27
90-day mortality (*n*, %)	214 (5.4)	160 (4.3)	0.011 (0.001–0.021)	0.019
ICU admission duration (days)	5.1 ± 7.7	4.6 ± 7.2	-	0.005
Mechanical ventilation duration (h)	47.3 ± 106.6	41.6 ± 102.3	-	0.018
CVP avg (mmHg)	8.1 ± 2.7	7.7 ± 2.0	-	<0.001
CVP max (mmHg)	12.0 ± 7.2	11.4 ± 7.8	-	0.005
CVP min (mmHg)	4.8 ± 2.8	4.6 ± 2.3	-	0.031

*Quantitative data are expressed as the mean ± SD. Qualitative data are expressed as n (%)*.

## Discussions

To the best of our knowledge, this is the first real-world study to evaluate the effects of QC targets in the ICU on mortality compared with conventional treatment. The 72 h average SpO2, Pmean, FiO2, PaO2, and VT values were significantly lower and the average PaCO2 was higher after QC than before QC. Lower 90-day mortality, less discharge against medical advice, and reduced durations of ICU admission and mechanical ventilation were found after QC compared with before QC. Interestingly, CVP was significantly lower in the QCA group compared with the QCB group.

Hyperoxia in critically ill patients can prolong their hospital stay and increase the ICU admission rates and mortality. A retrospective study including 3,322 patients receiving ventilator therapy showed that the first 24 h of FiO2 was positively linearly associated with mortality during hospitalization (12). A study with 4,459 patients with a median PaO2 of 231 mmHg admitted to the ICU after cardiopulmonary resuscitation from 120 hospitals showed that PaO2 increased the risk of death by 24% for every 100 mmHg increase, and an abnormally high PaO2 had a dose-dependent effect on hospital mortality (13). A meta-analysis revealed that hyperoxia may be associated with increased mortality in critically ill patient subsets, such as those with stroke [OR = 1.23 (1.06 to 1.43)] and traumatic brain injury [OR = 1.41 (1.03 to 1.94)], and those resuscitated from cardiac arrest [OR = 1.42 (1.04 to 1.92)] (14). Another meta-analysis also showed that hyperoxia group of critically ill patients with mechanical ventilation treated in the ICU included those with cardiac arrest, brain trauma, brain stroke, cardiac surgery, etc. had poor outcomes and increased mortality compared with the normal arterial oxygen group (15). Recently, Girardis et al. divided critically ill patients into conservative oxygen therapy (PaO2:70–100 mmHg or SpO2: 94–98%) and conventional oxygen therapy groups (PaO2 > 150 mmHg or SpO2: 97–100%). The results confirmed that ICU mortality in the conservative oxygen therapy group was significantly lower than that in the conventional oxygen therapy group (11.6% vs. 20.2%, *P* = 0.01). Moreover, the incidence of shock, liver failure, and bacteremia in the conventional oxygen therapy group was higher than that in the conservative oxygen therapy group (16). The concept of “target oxygen therapy” has been proposed and gradually accepted and promoted in the clinic (17). However, the guidelines in different countries have different definitions and requirements (17, 18). When the arterial oxygen saturation (SaO2) is 100%, PaO2 can reach 100–500 mmHg based on respiratory physiology. Clinically, the changes in SaO2 can be dynamically monitored by SpO2. Therefore, a SpO2/SaO2 value of 100% is very likely to cause hyperoxia. In the daily ICU setting, SpO2≠100% can be used as an easy and intuitive oxygen target. In our study, it was confirmed that SpO2≠100% can significantly lower the FiO2% and PaO2, which contribute to critically ill patient outcomes.

Low tidal volume (6–8 ml/kg) is a requirement for lung protection. Hypercapnic acidosis is a common phenomenon as the by-product of a protective lung ventilation strategy. Several studies revealed that varying degrees of permissive hypercapnia can decrease the mortality of severe ALI/ARDS patients (19–21). The CO2 protection against VILI has been supported by laboratory data (22, 23). The main reasons for this protection can be elucidated as CO2 influencing pulmonary gas exchange, decreasing shunting, and increasing arterial oxygenation (24). Here, PaCO2 40 mmHg was used as a target to achieve lung protection. In the clinic, ventilation parameters were adjusted, and patient sedation or even muscle relaxants were adopted to achieve lung ventilation. In this study, the average and minimum values of PaCO2 increased significantly after QC due to decreases in the tidal volume and respiratory rate. Of course, it is not necessarily true that higher values of PaCO2 are better. Our data showed that the maximum value of PaCO2 was well-controlled, and there was no difference before and after QC (44.4 ± 9.2 vs. 44.6 ± 8.2, *P* = 0.182).

To avoid tidal stretching, Pplat was maintained within 30 cmH2O. Pplat measurement always requires an inspiratory pause test, which is subject to many other factors, such as spontaneous breathing and intrinsic PEEP. Pmean reflects the actual mean alveolar pressure throughout the respiratory cycle (25) and can be influenced by intra-abdominal pressure (26). It can be easily read from the ventilator and recorded and adjusted in real time. In our previous study, Pmean was an independent risk factor for poor outcome (OR = 1.352, 95% CI, 1.288–1.419) (9). The cutoff value for Pmean for the prediction of 28-day mortality was 9.64. Therefore, after QC (from July 2015), Pmean 10 cmH2O was adopted as a target as the Pplat surrogate. The data from our study also demonstrated a lower Pmean after QC. A lower Pmean not only achieves the purpose of lung protective ventilation but also achieves the purpose of circulatory protective ventilation (10). Pmean QC could avoid pulmonary hypertension and right heart dysfunction caused by excessive airway pressure (9).

From the outcome data, we can see that although ICU mortality did not significantly differ, discharge against medical advice and 90-day mortality significantly declined. In particular, the ICU admission day and mechanical ventilation time significantly decreased after QC. The reason for this is based on the following: (1) the avoidance of hyperoxia of the lung itself; (2) the achievement of protective lung ventilation; and (3) the achievement of circulatory protective ventilation. Interestingly, CVP was decreased after QC. This is strong evidence that these QC targets may benefit the protection of the circulation.

CVP is considered reflective of right ventricular preload and is often used as a surrogate index for IV fluid challenge in critically ill patients for the maintenance of hemodynamic stability. Recently, an increasing number of studies have revealed that CVP may carry prognostic value. We found that lower CVP (<8 mmHg) in septic shock patients was associated with better end organ function and improved 28-day survival, and higher levels of central venous pressure in critically ill patients were associated with a worse prognosis and organ function (27–29). In our study, the CVP significantly decreased after QC. These three QC targets lower the CVP via the following mechanisms: (1) hyperoxia can significantly increase systemic vascular resistance, ventricular filling pressure, and pulmonary capillary wedge pressure to reduce cardiac output and stroke volume, which affect the diastolic function of the heart (30, 31). (2) The effect of elevated PaCO2 and hypercapnia on the heart and vascular smooth muscle is to reduce contractility and increase sympathetic activity and cardiac output (32). A high PaCO2 is also harmful. (3) A lower Pmean may reduce the effects of thoracic pressure on the right heart and pulmonary circulation (9).

Several limitations must be acknowledged. This was a single-center study that used a database to reveal the effects of QC targets used in the ICU setting. Although this study had a large sample size, it is still not possible to rule out the influence of different treatments on the prognosis of patients. In future studies, we should use a multicenter, multinational database to identify the QC targets that can affect the prognosis of critically ill patients. Second, this was a study dealing with QC targets in the ICU setting, so we did not give clearly defined ranges for different targets and did not further conduct subtype analyses. The roles of SpO2, PaCO2, and Pmean should be studied separately, and different diseases may result in different specific optimal ranges. Third, treatment methods may have changed between the before and after QC period, becoming more advanced. The treatment part of the sepsis guidelines underwent no substantial changes after the 2012 update. Therefore, we do not believe that this influenced the conclusions. Prospective randomized clinical trials should also be conducted.

## Conclusions

A series of QC targets (SpO2≠100%, PaCO2≮40 mmHg, Pmean≯10 cmH2O) resulted in a better prognosis of critically ill patients. These targets may contribute to avoiding high oxygen hazards, protecting against lung injury, and improving circulatory function.

## Data Availability Statement

The data used to support the findings of this study were provided by XW and DL under license and thus cannot be made freely available. Access to these data will be considered by the author upon request, with permission of the Department of Critical Care Medicine, Peking Union Medical College Hospital. Requests to access these datasets should be directed to DL (dwliu98@163.com) and XW (icuting@163.com).

## Ethics Statement

The studies involving human participants were reviewed and approved by The Institutional Research and Ethics Committee of the Peking Union Medical College Hospital approved this study for human subjects. The patients/participants provided their written informed consent to participate in this study. Written informed consent was obtained from the individual(s) for the publication of any potentially identifiable images or data included in this article.

## Author Contributions

LS and XW designed the study. PP and QZ collected and analyzed the data. PP wrote the paper. YL and DL critically revised the manuscript for important intellectual content. DL and XW gave final approval of the version to be published. All of the authors read and approved the final manuscript.

### Conflict of Interest

The authors declare that the research was conducted in the absence of any commercial or financial relationships that could be construed as a potential conflict of interest.
